# Comparative Transcriptomic Analysis of Multiple Cardiovascular Fates from Embryonic Stem Cells Predicts Novel Regulators in Human Cardiogenesis

**DOI:** 10.1038/srep09758

**Published:** 2015-05-21

**Authors:** Yang Li, Bo Lin, Lei Yang

**Affiliations:** 1 Department of Developmental Biology, University of Pittsburgh School of Medicine, 530 45th Street, Rangos Research Center, Pittsburgh, PA 15201.

## Abstract

Dissecting the gene expression programs which control the early stage cardiovascular development is essential for understanding the molecular mechanisms of human heart development and heart disease. Here, we performed transcriptome sequencing (RNA-seq) of highly purified human Embryonic Stem Cells (hESCs), hESC-derived Multipotential Cardiovascular Progenitors (MCPs) and MCP-specified three cardiovascular lineages. A novel algorithm, named as Gene Expression Pattern Analyzer (GEPA), was developed to obtain a refined lineage-specificity map of all sequenced genes, which reveals dynamic changes of transcriptional factor networks underlying early human cardiovascular development. Moreover, our GEPA predictions captured ~90% of top-ranked regulatory cardiac genes that were previously predicted based on chromatin signature changes in hESCs, and further defined their cardiovascular lineage-specificities, indicating that our multi-fate comparison analysis could predict novel regulatory genes. Furthermore, GEPA analysis revealed the MCP-specific expressions of genes in ephrin signaling pathway, positive role of which in cardiomyocyte differentiation was further validated experimentally. By using RNA-seq plus GEPA workflow, we also identified stage-specific RNA splicing switch and lineage-enriched long non-coding RNAs during human cardiovascular differentiation. Overall, our study utilized multi-cell-fate transcriptomic comparison analysis to establish a lineage-specific gene expression map for predicting and validating novel regulatory mechanisms underlying early human cardiovascular development.

Early heart formation is a stepwise process, including the consecutive differentiation of mesoderm, cardiac progenitor, and the terminal specification of cardiovascular lineage cells^1^,^2^,^3^. Key genes, which exhibit temporal and/or cell-type specific expression patterns could play essential roles in maintaining specific cell fates, as well as in reprograming differentiated cells back to pluripotency or to other types of cell fates. For example, overexpression of four embryonic stem cell (ESC) specific factors, Octamer-binding transcription facor 4 (OCT4), MYC, (Sex-determining region Y)-box 2 (SOX2) and KLF4, can reprogram fibroblasts into pluripotent stem cells^4^,^5^. The re-introduction of cardiac-specific factors, Gata4, Mef2c and Tbx5 converted mouse fibroblasts into induced cardiomyocytes both *in vitro* and *in vivo*^6^,^7^. Therefore, identifying lineage-specific genes, as well as their regulatory networks, could benefit the prediction and identification of novel genes and mechanisms that are essential in human cardiogenesis and human cardiovascular diseases.

Currently, most of the organ-specific genes in mammalians have been identified at the tissue-specific level. However, it is very difficult to obtain purified populations of specific cell types directly from early stage human embryo. With the recent advances in stem cell biology, the majority of human somatic cell types could be differentiated from human embryonic stem cells (ESCs) or induced pluripotent stem cells (iPSCs)^2^. This provides a unique access to the purified populations of cell lineages of interest at different differentiation stages. Using ESCs as an *in vitro* model to study early human heart formation, gene expression profiles of ESC derived cardiomyocyte-like cells have been extensively studied^8^,^9^,^10^,^11^,^12^. However, most of previous reports were focused on the differentially expressed gene in ESCs vs. a single type of terminally differentiated cell fate, beating cardiomyocytes (CMs). Noticeably, a recent study showed that during cardiac differentiation in human ESCs, cardiac regulatory genes, most of which are transcriptional factors, have distinct dynamic patterns of histone modifications from the CM-specific structural sarcomeric genes, indicating that combined analysis of histone modification dynamics plus gene expression profiles could be used to predict regulatory genes in early human CM development^13^. However, this study utilized a hESC-derived heterogeneous population to represent the committed stage of CMs, which contained non-CM cells. Therefore, genes specifically enriched in major cardiovascular lineages, including cardiomyocytes (CMs), smooth muscle cells (SMs) and endothelial cells (ECs), could not be distinguished and predicted by using a single lineage comparative analysis.

Recently, we established a new method for simultaneously enriching multipotential cardiovascular progenitor cells (MCPs), as well as MCP-specified CMs, SMs and ECs with a high purity from human pluripotent stem cells^14^. MCPs represent the earliest heart progenitor cells during human heart development. Access to MCPs allowed us to investigate two key events in early human heart formation, which are the induction of cardiovascular progenitors from pluripotency and the specification of cardiovascular lineages from the common progenitors. In this study, we performed deep-transcriptome sequencing (RNA-seq) of hESCs, MCPs, CMs, SMs and ECs, which represent pluripotency, multipotency and lineage-specification stages of early human heart formation, respectively. Analysis of the sequenced genes could profile temporally expressed genes (ESC**→**MCPs**→**CMs or SMCs or ECs) and genes with lineage-specific expression patterns (CMs vs. SMCs vs. ECs). In order to distinguish those lineage-enriched-genes (LEGs) from the genes with the relatively mild expression changes, we developed a new algorithm, GEPA, which could obtain single-lineage or multiple-lineages enriched-pattern of every single gene in all cell samples. Using optimized parameters, cardiovascular LEGs were identified at low false positive and false negative rates. Biological function enrichment of the lineage-specific LEGs modeled and revealed the functional characteristics of individual cardiovascular lineage. We found our GEPA predictions captured ~90% of top-ranked cardiac regulatory genes that were previously predicted based on their chromatin signatures in human ESCs^13^, indicating that our analysis could predict novel cardiovascular regulatory genes. We validated the critical role of ephrin/ephrin signaling pathway in human iPSCs, which was predicted by GEPA to regulate CMs differentiation from human iPSCs. Furthermore, using RNA-seq plus GEPA workflow, we identified dynamic changes of RNA splicing isoforms and lineage-enriched lncRNAs during human cardiovascular differentiation. All the results demonstrate that the cardiovascular cell resources and multi-cell-fate comparison algorithm could allow us to uncover and validate novel regulatory mechanisms during early human cardiovascular development.

## Results

### Deep-transcriptome sequencing of the five cell types representing different stages and cell fates of human cardiovascular development

**Figure 1:**
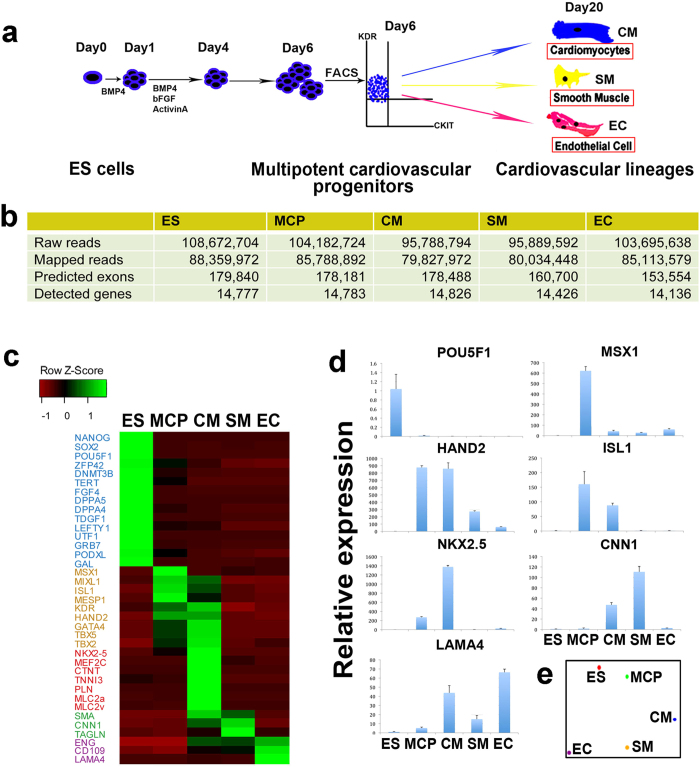
Deep-transcriptome sequencing of purified cardiovascular lineages differentiated from hESC. (**a**) Schematic representation of the sampling during cardiovascular differentiation from hESCs. (ES: embryonic stem cell; see other abbreviations in the main text; MCP, multipotential cardiovascular progenitors; CM, cardiomyocytes; SM, smooth muscle cells; EC, endothelial cells). (**b**) Summary of the deep-sequencing results. (**c**) Expression of the lineage marker genes quantitated by the normalized FPKM. Types of markers are color-coded. Blue: pluripotency markers; dark orange: mesoderm and early cardiac markers; red: cardiomyocyte markers; green: smooth muscle cell markers; purple: endothelial cell markers. (**d**) qRT-PCR validation of the marker gene expression. (**e**) Principle component analysis using FPKM of all the sequenced genes of the 5 cell types.

To characterize the genome-wide gene expression changes during human cardiovascular development, human RUES2 ES cells, RUES2-derived MCPs, as well as MCP-derived CMs, SMs and ECs, were enriched with a high purity (>90%) using our previously described method (Fig. 1a)^14^. We used RNA-seq to characterize the transcriptomes of the five cell types because RNA-seq has higher dynamic range in reporting the low-abundance transcripts than regular microarray analysis^15^. Paired-end RNA-seq using Illumina platform yielded nearly 100 million reads for each cell sample. A similar number of expressed genes were detected in each of those 5 cell types, ranging from 14136 in ECs to 14826 in CMs (Fig. 1b). Firstly, we examined the expressions of known lineage-specific signature genes quantified by RNA-seq (Fig. 1c). Pluripotency marker genes, such as Nanog Homeobox (NANOG), POU5F1 (OCT4), and SOX2 were exclusively expressed in hESCs. Early cardiac marker genes such as MESP1, ISL1, MIXL1and MSX1, were highly specific in MCPs and expressed with decreased levels in the CMs, SMs and ECs. Expression of other cardiac-related maker genes, including GATA4, TBX5 and HAND2, were detected from MCPs and specifically enriched in CMs, but not found in SMs or ECs. As expected, expression of CM-specific sarcomeric genes, such as CTNT, TNNI, MLC2V and MLC2A, were exclusively observed in CMs. SM or EC-specific genes were also identified by RNA-seq (Fig. 1c). The lineage-specificity from RNA-seq was further validated by qRT-PCR (Fig. 1d and Supplementary Fig. 1). Next, we performed principle component analysis (PCA) of all sequenced genes from the five samples (Fig. 1e). PCA revealed that the whole gene expression signature of MCPs was close to that of hESCs and far from those of the specified cardiovascular lineages, indicating a temporal change of whole transcriptional signatures during cardiovascular differentiation. In addition, PCA revealed that the whole gene expression signatures of SMs and ECs were more distantly related to that of MCPs than CMs (Fig. 1e), suggesting a significant transcriptome change occurs during the early segregation of CMs and vascular cells from the same progenitors. Hierarchical clustering of the five transcriptomes also demonstrated a similar result (Supplementary Figure 2) as PCA. Overall, using our new cardiovascular cell resources, the gene expression profiles by RNA-seq defined the pluripotency, multipotency and specification stages during early human heart development, which allowed us to conduct temporal and comparative analysis of multiple cell fates in human cardiovascular development.

### GEPA algorithm identified lineage-enriched genes during human cardiovascular development

**Figure 2:**
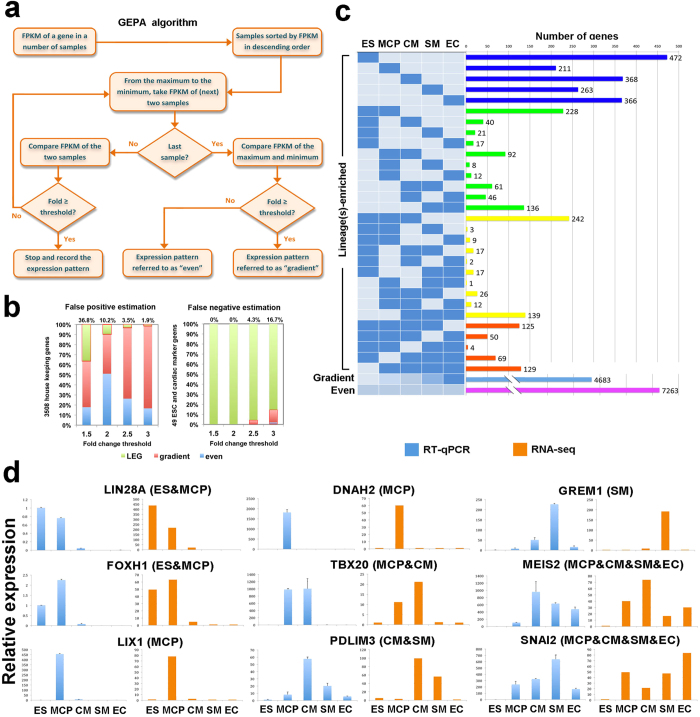
GEPA algorithm identified lineage-enriched genes from RNA-seq data. (**a**) Schematic representation of the “GEPA” algorithm workflow to identify the lineage-enriched patterns of the genes. (**b**) Estimation of false positive and false negative ratio of GEPA algorithm at different thresholds of FPKM fold change. (**c**) Distribution of the genes across the expression pattern categories. Lineage-enriched patterns are indicated at left side. In the same row, rectangles filled with blue are at least 2.5 fold higher than those in light gray. The bars indicating the number of genes are color coded. Blue for single lineage-enriched groups. Green, yellow and orange for two, three and four lineage-enriched groups, respectively. Light blue for “Gradient” group and purple for “Even” group. (**d**) qRT-PCR validation of the signature genes for lineage-enriched categories. Gene name and expression pattern defined by GEPA (in brackets) were shown above the plots.

To compare the relative expression levels of each gene within the five cell types, we developed a new algorithm named Gene Expression Pattern Analyzer (GEPA) (Fig. 2a). This algorithm recognized the single or multiple lineages enrichment pattern (LEP) of each individual gene within the five cell types and the lineage-specificity was set based on the fold change of gene expression over an arbitrary threshold (see Methods for details). Using GEPA, all sequenced genes from the five cell types could be grouped based on their lineage-specificity. If a clear lineage-specific enrichment was not identified, genes would be grouped into “Gradient” or “Even” categories, which indicate the patterns of commonly expressed genes across all five samples with mild or no lineage specificity, respectively (Fig. 2a). Since LEP of a single gene is largely relying on the arbitrary setting of fold change thresholds, to estimate the false positive recognition of the LEP by GEPA, we input 3804 documented human housekeeping genes as negative controls to run GEPA under four fold change thresholds, 1.5, 2, 2.5 and 3^16^. Because the 3508 housekeeping genes should not exhibit lineage-specificity and should be grouped into “Gradient” or “Even” categories, the less ratio of LEP from the 3508 genes would indicate a higher definition to recognize LEP. We found that false positive rates of those housekeeping genes were much lower with thresholds of 2.5 and 3 (3.5% and 1.9% respectively) when compared to the thresholds of 1.5 and 2 (36.8 and 10.2 respectively) (Fig. 2b). Next, we input 49 embryonic stem cell-specific and cardiac-specific genes as positive controls to run GEPA and calculated the false negative rates (Supplementary Table. 1). Thresholds 1.5 and 2 captured all the positive controls with LEG, whereas thresholds of 2.5 and 3 lost LEG in 4.3% and 16.7% of all inputs, respectively. All these tests indicated that thresholds of 1.5 and 2 were too loose and threshold of 3 was too stringent. However, threshold of 2.5 kept a high definition for LEG recognition without introducing too much falsely identified LEG (Fig. 2b). Thus, we then classified all sequenced genes from the five cell types into 32 categories using a threshold of 2.5 (Fig. 2c). Of all sequenced genes, a total of 79% genes exhibited no lineage(s)–enrichment, with 48% in the “Even” category and 31% in the “Gradient” category. Approximately 21% sequenced genes showed single or multiple lineages-enrichment. In addition, we found the categories of single lineage-enrichment contained a higher average number of genes per category (total 1680 genes in 5 categories) than that of categories with a multiple-lineages specificity (total 1560 genes in 25 categories) (Fig. 2c). The genes showing multiple-lineages-specific enrichment were mainly distributed in categories of “ES&MCP”, “ES&MCP&CM”, “SM&EC”, and “CM&SM&EC”. This lineage enrichment analysis was consistent with the PCA and hierarchical clustering results in Fig. 1e and Supplementary Fig. 2, indicating that the more closely related cell types during cardiovascular differentiation would share the more commonly expressed genes (Fig. 2c). The LEG distribution pattern was lost when the sequenced genes of 5 cell types were shuffled (Supplementary Table. 2), indicating the LEG distribution could genuinely recognize the intrinsic nature of gene expressions in those cell types. Noticeably, the LEG patterns from RNA-seq results were highly consistent with the qRT-PCR validation in Fig. 2d. Therefore, our newly developed GEPA algorithm successfully identified LEG of all the sequenced genes for studying human cardiovascular development.

### Lineage-enriched genes uncovered functional specialization/transition during human cardiovascular development

**Figure 3:**
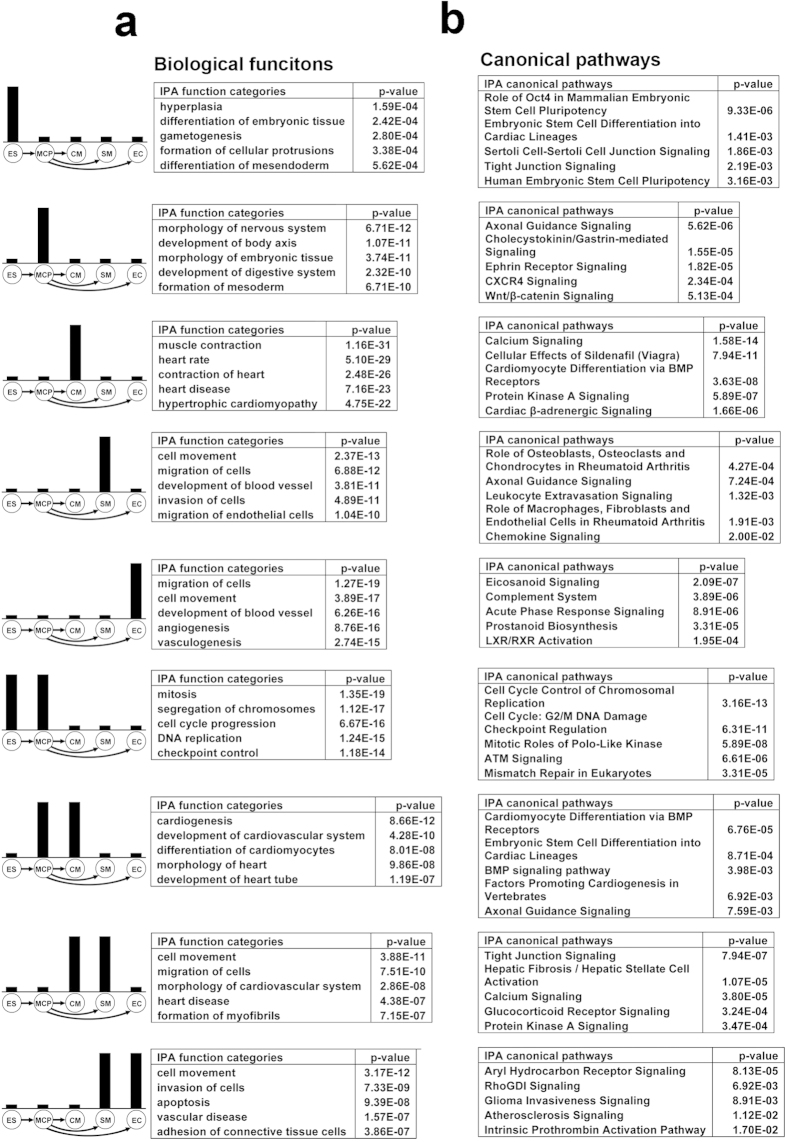
During cardiovascular differentiation, both single and multiple lineage-enriched genes showed enrichments in specific biological functions or canonical pathways. The lineage specificities of gene groups are illustrated as bar charts (left). Bar lengths are indicative of lineage specificity, not showing the actual expression levels. (**a**) Ingenuity biological functions enrichment of the single- and dual-lineage-enriched gene groups. (**b**) Ingenuity canonical pathways enrichment of the single- and dual-lineage-enriched gene groups.

During heart development, cardiac specific genes are expressed in a temporal manner to control the sequential differentiations of mesoderm, cardiac progenitor, and finally the commitment of cardiovascular lineages. At each specific differentiation stage or individual lineage, a group of LEGs could account for the time-specific and lineage-specific functions. Therefore, we performed Ingenuity IPA analysis to understand biological functions, which are determined by the LEGs. IPA revealed that LEGs were strongly enriched into distinct biological functional categories and canonical pathways (Fig. 3). For example, hESC-enriched genes were involved in functional categories related to differentiation of embryonic tissues and differentiation of mes/endoderm, and most MCP-specific genes were enriched into the functional categories of embryonic development (development of mesoderm, body axis, reproductive organ, digestive organ and morphology of nervous system etc.) (Fig. 3a). These results reflect the multi-potential and plasticity of MCPs. Genes with specific enrichment in terminally differentiated CMs were mostly responsible for cardiac functions, such as cardiac muscle contraction, heart rate, and hypertrophic cardiomyopathy (Fig. 3a). EC-enriched genes were mainly related to the development of blood vessel and movement of cells, which is consistent with the known functions of ECs^17^. The SM-specific genes were enriched into similar biofunctional categories as EC enriched genes, indicating a close interaction of these two vascular cell types in the formation of blood vessels during cardiogenesis (Fig. 3a).

Interestingly, same as the single-lineage-enriched genes, the multi-lineages-enriched genes showed specific functional enrichments as well. Genes involved in cell cycle progression, mitosis related functions were enriched in “ES&MCP”, whereas genes enriched in “MCP&CM” were involved in biological functions related to cardiogenesis, including development of heart tube, differentiation of cardiomyocytes and morphology of heart (Fig. 3a). These indicate the overall shift of a gene regulatory network from controlling cell proliferation to cardiovascular differentiation. Genes enriched in “EC&SM” were involved in vascular and connective tissue formation related functions, but the genes enriched in “SM&CM” were concentrated into the biofunctions of formation of myofibrils and heart disease.

Similarly, canonical pathways also showed specific enrichment of the LEG groups. For example, stem cell pluripotency in “ES”; Wnt/β-catenin signaling in “MCP”; calcium signaling in “CM” and “CM&SM”; eicosanoid signaling in “EC”; BMP signaling in “MCP&CM” (Fig. 3b). In addition, we observed that key component genes of some signaling pathways, which are known to play important roles in cardiovascular development, such as Wnt/β-catenin, BMP and Nodal, exhibited temporal and lineage-specific enrichments (Supplementary Figure 3). Taken together, these results suggest that LEGs underlie the dynamic biofunctional specializations and the temporal activations of signaling pathways to control the stepwise development of cardiovascular lineages from pluripotent ESCs. Moreover, comparisons of biological functions that were enriched by cardiovascular single-lineage LEGs vs. multiple-lineages LEGs could further define cardiac muscle vs. vessel related functional differences.

### Lineage-specific transcription factor network analysis modeled the temporal development of cardiovascular lineages in human ESCs

**Figure 4:**
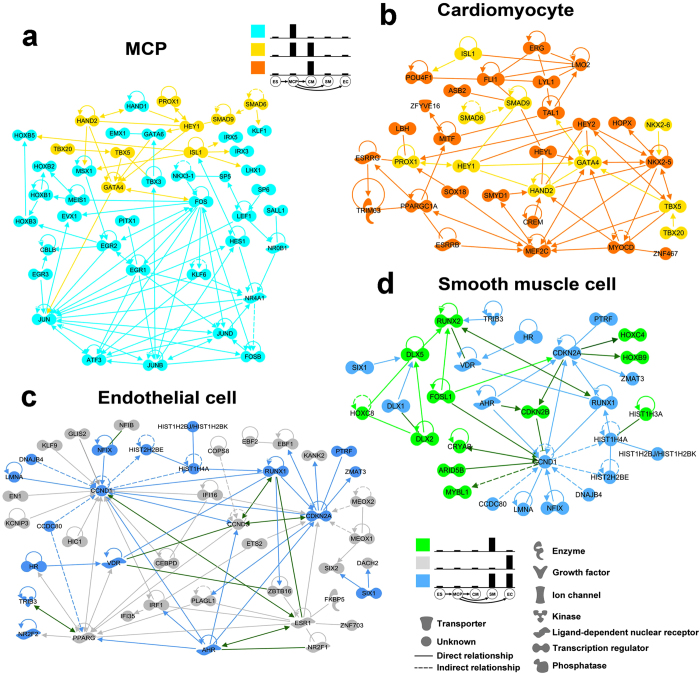
Network connections of lineage-enriched transcription factors in different cardiovascular cell types. LEG groups are color-coded and lineage-specificity was shown using the same bar charts as in Fig. 3. The connection of transcription factors was built based on IPA (Ingenuity Pathway Analysis, Ingenuity Systems). (**a**) Multipotential cardiovascular progenitor. (**b**) Cardiomyocyte. (**c**) Smooth muscle cell. (**d**) Endothelial cell.

Transcription factors are interconnected thus forming networks to specify lineage commitment and cellular function in a temporal manner^18^. Thus, we sought to dissect the early process of human cardiovascular development by analyzing the transcription factor networks (TFNs) using the Lineage-Enriched Transcription Factors (LETFs). It has been previously demonstrated that NANOG, SOX2, OCT4 are among the central TFNs in hESCs^19^,^20^ and we observed the same result by using Ingenuity IPA to analyze the ES specific TFs (data not shown). Additionally, we built the lineage-specific TFNs in MCP, CM, SM and EC (Fig. 4). TFNs of each single lineage indicated the dominant gene programs controlling lineage-specific biofunctions, whereas the TFN of “MCP&CM” reflected the dynamic TFN transition during cardiovascular development and TFN of “SM&EC” revealed commonly shared mechanisms during vascular cell formation. For example, we found the MCP-specific TFN was centered by jun proto-oncogene (JUN), FOS and related genes (JUNB, FOSB, JUND, ATF3 and EGR genes) (Fig. 4a), indicating the high proliferative potential of MCPs as previously described^21^. Sub networks of TFNs in cardiac (such as HAND1/2, GATA4 and ISL1) and hematopoietic (such as the HOXB genes) developments were also present in MCP, indicating the plasticity of the MCP in its differentiation potential. The expression level of some cardiac specific TFs, such as HAND2 and GATA4, remained high in both “MCP” and “CM” (Fig. 4a, b, yellow color labeled genes), indicating that these transcription factors may function at stages of both heart progenitor formation and CM specification. We term these early onset TFs as “early” cardiac TFs. Transcriptions of CM-specific cardiac TFs, such as MEF2C and NKX2.5, were initiated during the differentiation from MCPs to CMs and were maintained high in CM, indicating their vital role during CM fate specification (Fig. 4b). We term these late onset TFs as “late” cardiac TFs. Therefore, the dynamic transitions of TFNs from the proliferation-controlling TFs in MCP (Blue genes in Fig. 4a) to “early’ cardiac TFs (Yellow genes in Fig. 4a,b) and subsequently to “late’ cardiac TFs (Orange genes in Fig. 4b) revealed the underlying programs controlling human CM development. Interestingly, we found MEF2C, GATA4 and HAND2 centralized the TFNs of MCP and CM, which is consistent with their essential roles in directly reprogramming fibroblasts into cardiomyocytes (Fig. 4b)^6^,^7^,^22^. Almost no TFs were co-enriched in MCP&SM or in MCP&EC, indicating a more profound transcriptional change during the early segregation of MCP towards vascular cell fate than to cardiac muscle fate. However, many TFs are enriched in both SM and EC, such as cyclin-dependent kinase inhibitor 2A (CDKN2A), CCND1 and RUNX1 (Fig. 4c,d), indicating the common regulatory mechanism during vasculogenesis. Therefore, TFNs, which were built by the LETFs, could globally reveal the temporally transcriptional and biofunctional changes during human cardiovascular development.

### GEPA predicted novel regulators during human cardiovascular development

**Figure 5:**
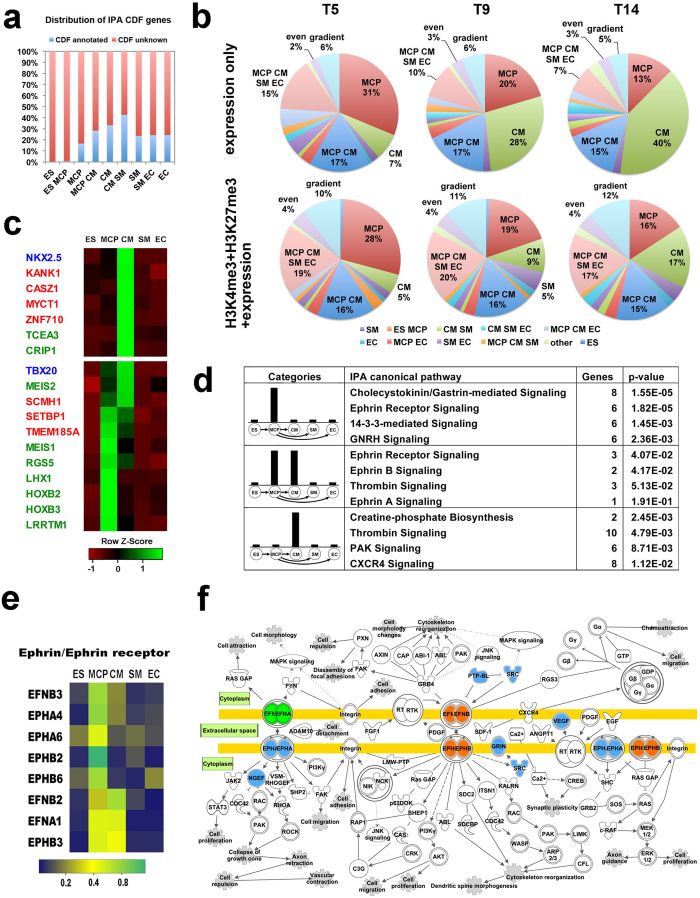
LEGs identified by GEPA predict novel regulatory genes and pathways in human cardiovascular differentiation. (**a**) Percent of the genes with or without annotation in “cardiovascular development and function” from Ingenuity knowledge database in the LEG groups identified by GEPA. (**b**) Our GEPA analysis of top 100 novel cardiac regulatory genes previously predicted by Paige *et al.*^13^ when considering “expression only” or “H3K4me3+H3K27me3+expression “ at T5, T9 and T14 of CM differentiation, respectively. (**c**) Examples of predicted functional genes by GEPA. Dynamic expression of these genes is shown in heatmap. Known cardiac regulators are highlighted in blue. Our predicted candidates, which are overlapping with chromatin dynamics-based predictions by Paige *et al.* and Wamstad *et al*., are shown in green^13^,^38^. Novel regulatory genes solely predicted by GEPA are labeled in red. (**d**) Predicted novel regulatory pathways in cardiovascular differentiation using GEPA and Inginuity IPA pathway enrichment analysis. (**e**) A heat-map showing the lineage-specific expression pattern of ephrin and ephrin receptor genes during cardiovascular differentiation. To indicate the lineage-specificity, the relative gene expression was shown as percent in the sum of all the cell types in the heatmap. (**f**) Illustrative Ephrin/Ephrin signaling pathway imposed on a pathway map based on Ingenuity IPA showing localizations of the LEGs. Color code of the molecule indicates its lineage specificity. Blue indicates enrichment in “MCP” ; Orange, both “MCP” and “MCP&CM”; Green, “MCP&CM”.

Based on the gene function annotation from Ingenuity knowledge database, the cardiovascular specific LEGs contain up to 40% known cardiovascular functional genes and, as expected, no cardiac regulatory genes were found in the ES-specific LEGs (Fig. 5a). Over 60% genes with unknown functions in heart development were found in our cardiovascular specific LEGs, implying the existence of potentially novel regulators within those genes. To test if GEPA could predict novel cardiac regulatory genes, we next analyzed the lineage specificities of a list of previously predicted cardiac regulators during cardiac differentiation in human ESCs. By comparing with our list of GEPA defined LEGs, the lineage-specificities of top 100 cardiac genes, which were previously ranked by using “expression only” or “H3K4me3+H3K27me3+expression” criteria at each of three time points T5, T9 and T14 of CM differentiation in that study, were distinguished, respectively (Fig. 5b)^13^. This allowed us to test whether GEPA analysis of this study could consistently recapitulate early dynamics of cardiovascular development in human ESCs, and whether GEPA analysis could further distinguish the lineage specificity of those novel cardiac genes predicted by other research laboratories. As shown in Fig. 5b, of the top 100 genes predicted by the “expression only” or “H3K4me3+H3K27me3+expression” criteria, 8 ~ 9% or 14 ~ 15% falls into non-lineage specific “Gradient” or “Even” categories, respectively. This indicates that our GEPA filtered approximately 10 ~ 15% non-lineage specific genes from previous predictions and recognized approximately 85 ~ 90% of the cardiac genes, which were predicted by considering both transcriptional activity and chromatin dynamic. In the previous study^13^, hESCs were sampled at T5, T9 and T14 of cardiac differentiation to represent the early, middle and late stages of CM differentiation. After the cardiovascular lineage specificity analysis using GEPA, we found approximately 30% of T5, 20% of T9 and 10% of T14 cardiac genes were enriched into MCP group, whereas an increased ratio of predicted regulatory genes from T5 to T14 were enriched into CM group, indicating the progressively CM commitment from pluripotency. Additionally, approximately 30% of previously predicted cardiac genes exhibited a multiple lineage enriched pattern, with around 10-20% of them in “ MCP&CM&SM&EC” and 15% in “MCP&CM”. The high incidence of multiple-lineage enrichments of previously predictions shows that the comparative analysis using a single lineage-CM differentiation in hESCs could not define the real CM-specific transcripts, given that CM/SM/EC share some commonly expressed cardiovascular-specific genes.

Next, we analyzed the list of genes that are specifically expressed in MCP or CM, which were predicted by both our GPEA and previous predictions (Fig. 5c). This gene list contained known cardiac specific genes (blue color labeled genes in Fig. 5c), as well as genes that were overlappingly predicted (green color labeled genes in Fig. 5c) and solely predicted by GEPA (red color labeled genes in Fig. 5c). All these predicted novel cardiac genes showed very similar expression patterns as the well-known cardiac regulatory genes, such as NKX2.5 and TBX20 (Fig. 5c). Although most of those predicted genes are transcriptional factors, some of them were found to be components of canonical signaling pathways, suggesting that those putative novel cardiac genes might regulate cardiovascular development via modulating signaling pathways. Interestingly, we found that eprin/ephrin receptor signaling pathway genes were highly specifically enriched in MCP and CM categories (Fig. 5d) and most of the LEG genes in the eprin/ephrin receptor signaling pathway genes encode ephrin or ephrin receptors (Fig. 5e,f). These analyses suggested that ephrin/ephrin receptor signaling might have unclear functions during cardiomyocyte specialization from human ESCs. Alltogher, these results demonstrate that GEPA could be applied to predict novel functional or regulatory genes in human cardiovascular development, as well as in other biological processes with multi-stages definitions.

### Validation of the GEPA predicted regulatory signaling pathways during cardiovascular differentiation of hiPSCs

**Figure 6:**
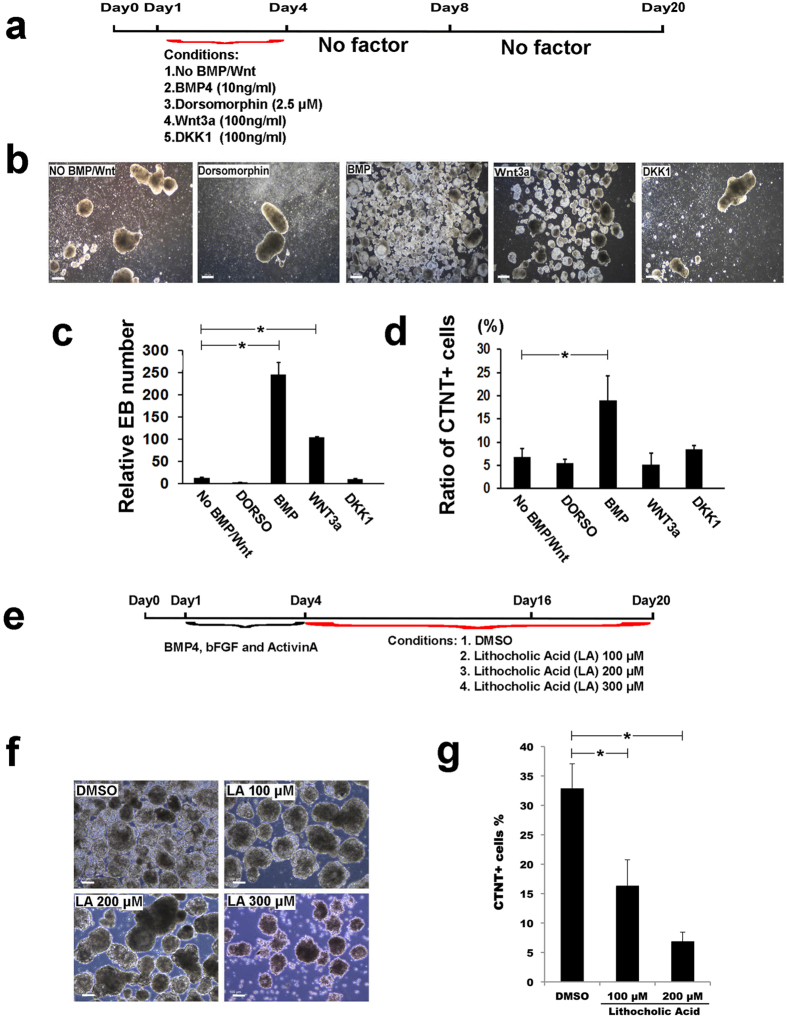
Validation of GEPA predicted regulatory pathways using *in vitro* cardiovascular differentiation from human iPS cells. (**a**-**d**) Validating the roles of well-known signaling pathways in human cardiovascular differentiation. (**a**) A scheme of modulating BMP and Wnt pathways from day1 to day4. No factor was added from day 0 to day 1 and ActivinA (3 ng/ml) was added into all conditions through day 1 to day 4. (**b**) Representative images showing EBs of day 20. Scale bar, 100 μm. (**c**) Quantitative analysis of EB numbers at day 20. Statistical analysis were performed with unpaired *t-*test, *p < 0.01 (**d**) Quantitative analysis of ratio of CM at day 20 EBs. EBs were dissociated, immunostained with anti-CTNT antibody and FACS analyzed. CTNT+ cells represent CMs. Statistical analysis were performed with unpaired t test, *p < 0.1 (**e**) A scheme of inhibiting ephrin receptor signaling pathway using lithocholic acid (LA), which is a small molecule inhibitor of ephrin receptors, from day 4 to day 20 of differentiation in human iPS cells. DMSO treatment served as control. (**f**) Representative images showing day 20 EBs in DMSO and LA treated wells. Scale bar, 100 μm. (**g**) FACS analysis of ratios of CTNT+ CMs from day 20 EBs. Results were represented as mean ± S.D., from 3 independent experiments, statistical analysis were performed with unpaired *t* test, *p < 0.01.

Since the findings from human ESCs could benefit the generation of patient-specific cardiac cells from human iPSCs, we next validated the functions of predicted signaling pathways using human iPSCs. Firstly, we examined the roles of several key signaling pathways in vertebrate heart development, including BMP, Wnt and FGF, during cardiovascular differentiation from human Y1-iPSCs (Fig. 6). We generated embryoid bodies (EBs) from Y1 iPS cells at day 0 using our established cardiac differentiation method (Fig. 6a) , followed with modulations of BMP and Wnt pathways before the formation of MCPs, which was from day 1 to day 4 of differentiation. Adding BMP4 (10 ng/ml) significantly increased the total number of EBs from day 4, when compared with the absence of BMP4 or with the adding of BMP antagonist, Dorsomorphin (2 μM). Activating Wnt pathway by adding Wnt3a (100 ng/ml) from day 1 to day 4 increased the number of EBs, whereas blocking Wnt pathway by adding a Wnt inhibitor, DKK1, substantially decreased the EB formation efficiency (Fig. 6b,c). Next, we changed all the day 4 media, which contained various pathway modulators (Fig. 6a), to basal medium without any factor until day 20. The day 20 EBs were dissociated, followed by immunostaining of CTNT, which is a marker for CM. Ratio of CMs (CTNT+ cells) was examined using fluorescence-activated cell sorting (FACS). Approximately 20% CTNT+ CMs were generated with presence of BMP from day 1 to day 4, which was significantly higher than that from the control condition containing no BMP/Wnt modulators from day 1 (Fig. 6d). Although early presence of Wnt3a increased the number of total EBs at day 4 (Fig. 6b,c), only 5% CM was found at day 20, which was similar to the control condition containing no pathway modulators (Fig. 6d). This indicates that Wnt pathway could play different roles in early vs. late stages of human cardiovascular development. Additionally, these data demonstrate that our cardiac differentiation system could provide an *in vitro* model for testing the functionality of signaling pathways in early human cardiovascular development. Therefore, we next sought to examine the role of ephrin receptor signaling pathway, which was highly enriched in the MCP formation stage by GEPA and IPA analyses (Fig. 3b and Fig. 5d) with unknown function during early human heart formation. The expressions of ephrin and ephrin receptor genes were highly enriched in “MCP” and “MCP&CM” (Fig. 5e), suggesting an essential role of ephrin receptor signaling during early human CM differentiation. A previous study indicated that ephrin activity could be completely blocked by adding lithocholic acid (LA) at 200 μM, which is a small molecule to inhibit binding of ephrin ligands to EphA/B receptors^23^. We thus added LA into the differentiating EBs from day 4 to 20 with increased doses (Fig. 6e) to examine the impact of blocking Ephrin pathway on human MCP formation and CM commitment. Treating with LA at 100 and 200 μM did not affect the growth of EBs, whereas the loading of LA at 300 μM showed negative effect in EB growth from day 6 (Fig. 6f). To determine the efficiency of CM differentiation from human iPS cell post modulating of ephrin signaling, we used FACS to measure the ratios of CTNT positive CMs in day 20 EBs and observed a dose-dependent suppressing role of LA in CM-derivation from iPS cells (Fig. 6g). Consistently, a lower ratio of beating EBs was found in the LA treated EBs when compared with the control DMSO treated EBs (Supplementary Video 1 and 2). These results, together with the finding that EphB4-knockout mouse ES cells were deficient in CM differentiation^24^, demonstrate that ephrin signaling plays a key role in mammalian cardiogenesis. Moreover, this demonstrates that the GEPA analysis of comparative RNA-seq data could successfully predict novel functional signaling pathways in early human cardiovascular development, and possibly in other biological processes.

### GEPA predicted splicing alterations associated with differentiation stages and lineage-enriched long non-coding RNAs during human cardiovascular differentiation

**Figure 7:**
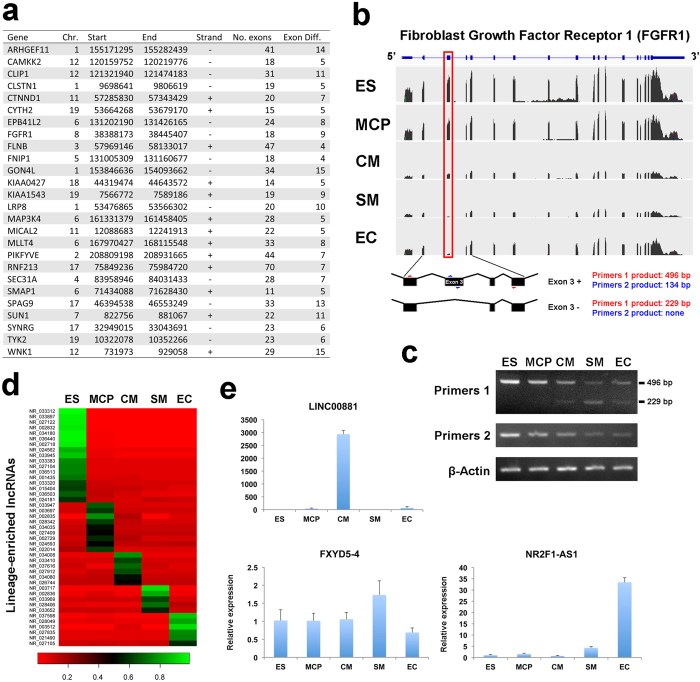
GEPA predicted differentiation-associated alternative RNA splicing and lineage-enriched long non-coding RNAs during human cardiovascular differentiation. (**a**) Genes with exon changes detected by RNA-seq during human cardiovascular differentiation. Locations of the genes in Hg18 human genome assembly were shown along with total exon number (No. exons) and number of exons with changes (Exon diff.). (**b**) Differential use of the exon 3 of FGFR1 during human cardiovascular differentiation. Stacks of sequencing reads in the five lineages were shown. Distances of the exons are NOT the actual intron lengths. Change in the expression of exon 3 was highlighted by a red frame. Primers to verify the expression of the exon 3 were indicated by red or blue arrows. (**c**) Semi-quantitative RT-PCR validation of the exon 3 usage during human cardiovascular differentiation. (**d**) Lineage-enriched long non-coding RNAs during human cardiovascular differentiation identified by GEPA. To indicate the lineage-specificity, the relative expression was shown as percent in the sum of all the cell types in the heatmap. (**e**) qRT-PCR validation of the lineage-enriched expressions of lncRNAs.

Alternative splicing produces diversities in protein output of the genome. It could play a role as regulatory mechanism in cell fate decision. A well established example is that the protein isoforms of FOXP1 gene produced by alternative splicing can have differential regulatory activities in maintaining pluripotency or promoting differentiation^25^. To identify the alternative splicing during human cardiovascular differentiation, we used GEPA to analyze the lineage-enrichment pattern of the splicing isoforms of the genes. We found a number of genes have differential expression patterns of the isoforms (Fig. 7a). Among them, a typical example is the differential usage of the exon 3 of Fibroblast Growth Factor Receptor 1 (FGFR1) gene during human cardiovascular differentiation (Fig. 7b). In early stage of differentiation (ES and MCP) sequencing reads of exon 3 are at similar levels as adjacent exons. However, exon 3 level is much lower compared to adjacent exons when the ES cells differentiated into SMs and ECs, in spite of an overall decrease of FGFR1 level in those cells (Fig. 7b). To validate the differential usage of exon 3 of FGFR1, we designed two primer pairs to detect the exon 3 present and exon 3 absent isoforms (Listed in Supplementary Table. 3). PCR using primers 1 produce a 496 bp product indicating the isoform with exon 3, or a 229 bp product without exon 3. PCR primers 2 locate in exon 3 and produce a 134 bp product only if exon 3 exists (Fig. 7b). Consistent with the RNA-seq, RT-PCR detected progressive decrease of FGFR1 isoform with exon 3 during the differentiation of hESC towards cardiovascular cell fates. FGFR1 isoform absent of exon 3 were not detectable in ES and MCP, while clearly detected in CMs, SMs and ECs (Fig. 7c). These results indicate the expression change of FGFR1 isoforms by skipping exon 3, which encode 89 amino acids, during the stage of MCP to cardiovascular lineage differentiation. This result suggests that our RNA-seq plus GEPA workflow could predict stage-associated splicing switch of genes during human cardiovascular differentiation.

Long non-coding RNAs (lncRNAs) are a group of transcripts without protein-coding capacity and with lengths greater than 200 nt. Many lncRNAs are expressed in specific tissues and involved in development. Recent studies showed that lncRNAs could play a role as key regulators in cardiovascular development^26^,^27^,^28^. In addition to the protein-coding genes, RNA-seq also detected lncRNAs. Since most regulatory lncRNAs involved in development are spatiotemporally specific, we applied GEPA to identify the lineage-enriched lncRNAs during human cardiovascular differentiation. A number of lncRNAs are identified to be specifically expressed in a single lineage during cardiovascular differentiation (Fig. 7d). Using qRT-PCR, we validated the expression pattern of some lineage-enriched lncRNAs (Fig. 7e). Among them, LINC00881 (NR_034008) is highly specific in cardiomyocytes and NR2F1-AS1 (NR_021490) is highly enriched in endothelial cells, indicating that these lncRNAs might play a role in the specification or functions of related cell types (Fig. 7e). These results suggest that our RNA-seq plus GEPA workflow is able to identify lineage-enriched lncRNAs, which may help to discover functional lncRNAs in human cardiovascular differentiation.

## Discussion

Early heart formation is precisely controlled by dynamic changes of underlying gene expression programs. Recently, mouse ES cell and human ES cells have been utilized as an *in vitro* model to study mammalian cardiogenesis^29^. However, most previous studies were focused on the differentiation of cardiomyocytes from ESCs, and the majority of previous comparative studies were conducted between ESCs and ESC-derived cardiomyocytes^8^,^10^,^30^. In this report, we differentiated and purified a population of early stage cardiovascular progenitor cells (MCPs) from hESCs, as well as specified the MCPs into three major cardiovascular cell types including CMs, SMs and ECs^14^,^31^. This novel cardiovascular differentiation system allowed us to model the early events and study the temporal cell fate transition from pluripotency to terminally differentiated cardiovascular cells. Additionally, the introductions of a key intermediate state of MCPs and two parallel vascular cell fates including smooth muscle cells and endothelial cells, significantly improved the accuracy of the lineage-specificity mapping of cardiovascular specific transcripts. In this study, we found that TFN of each single lineage underlies its dominant gene program to control the lineage-specific signature biofunction, whereas comparing the TFNs of temporally adjacent lineages could recapitulate the dynamic TFN transitions during cardiovascular development. For example, analyzing the changes of TFNs from ESCs→MCP→MCP&CM→CM could dissect the mechanisms underlying temporal CM development in hESCs. In addition, the multiple-lineage GEPA analysis could distinguish cardiovascular cell fate specificity of genes with a significantly increased resolution. For example, the CM-enriched genes could be distinguished from the genes enriched in CM&SM or CM&SM&EC groups. The refined lineage-specificity map may benefit the identification of key factors pertaining to specific temporal window(s) during differentiation, or to a specific cell fate.

Cluster analysis is the classic method to conduct multiple-state-comparing analysis^32^. Genes with similar expression patterns are clustered together and compared. In our study, we developed a new algorithm, which was named as Gene Expression Pattern Analyzer (GEPA), to analyze the gene expression pattern in the five lineages. GEPA is basically the combination of two widely used methods: sorting and binary comparison. It calculates expression pattern of each gene and doesn’t need comparisons between genes. GEPA can directly output transcriptional enrichment pattern of each single gene within multiple compared lineages without the need of further manual identification of clusters from original large data table. Instead, a simple application of sorting in a table processing software such as Microsoft Excel will group the genes with the same expression pattern together. Therefore, GEPA is more user-friendly for researchers without strong computational background. Because GEPA calculates the expression pattern for each gene one by one, it does not need much memory to run large set of data in parallel. Our data sets (15132 genes in five lineages) could be analyzed on commonly built PCs within a few seconds. Using our RNA-seq data sets, we got low false positive rates and false negative rates at the fold change threshold 2.5. However, the threshold of fold change can be manually adjusted to optimize the outcome stringency according to the false positive and false negative rates. Theoretically, there is no limit of the number of states (samples/lineages) for GEPA. In addition to the RNA-seq data, GEPA could be applied to comparative analysis of other data sets with comparable states, such as data from microarray and ChIP-seq etc. Therefore, GEPA could be widely useful for pattern analysis of multiple states/samples in a wide variety of biological studies.

What genes determine a distinct cell type is a long-standing question in developmental biology. With the refined lineage-specificity map in human cardiovascular differentiation, we found that majority (80 ~ 84%) of the genes are expressed at similar levels throughout the whole cardiovascular development process. The single lineage-enriched genes take up only 1.43 ~ 3.19% of all the detected genes in a certain cell type. Identification and comparison of single-lineage-enriched genes versus multi-lineage-enriched genes could distinguish gene groups underlying functional transitions during cardiovascular development. For example, in MCPs, we found high expression levels of genes related to mitosis, mesoderm development and cardiogenesis. Although ESCs express high level of mitosis related genes and CMs express high level of cardiogenesis related genes as well, the transient and simultaneous expressions of those genes in MCPs makes MCP unique in its plasticity, high proliferative potent and proclivity to differentiate into cardiomyocytes (Fig. 3). Our results suggest that the comparative cell-fate analysis may reveal combinatorial gene modules that determine unique cell types.

Determining the ontogenetic tree of cell lineages is important for understanding the developmental origin of a specific cell type. Using PCA and hierarchical clustering analyses, we found that CM was more correlated to MCP than to SM and EC during cardiovascular differentiation, indicating that the differentiation of MCPs towards vascular cells would require a more profound gene expression change than MCP differentiation to cardiac muscles (Fig. 1e and Supplementary Figure 2). This observation is consistent with the finding of more enriched genes in “MCP&CM” than those in “MCP&SM” or “MCP&EC” (Fig. 2b). On the other hand, SM and EC were found to share some core transcription factors (Fig. 3 and Fig. 4). All these results suggest the existence of a bi-potential vascular progenitor for SM/EC during human heart formation. However, further investigation is needed.

Cell-fate establishment often coincides with the expression of lineage-specific signature genes. A few of those key genes were shown to change cell identity. For example, induced pluripotency could be established by introducing four pluripotency marker genes into fibroblast, and functional neuron or cardiomyocytes could be transdifferentiation from fibroblast by overexpressing lineage-specific TFs^4^,^5^,^6^,^7^,^33^,^34^,^35^. Therefore, identification of key lineage-specific regulatory genes is of great significance for uncovering cell fate-determinant genes. Here, the LEGs, which were identified by GEPA from RNA-seq data, could predict novel regulatory genes and functional mechanisms in human cardiovascular development and fate-determination. Such capacity has been proved in this study by defining the lineage-specificity of a list of novel cardiac regulatory genes, which were previously predicted by considering both dynamic histone modification and gene expression changes during cardiac differentiation in human ESCs^13^. Additionally, we predicted putative function of ephrin pathway in CM differentiation from hESCs. This prediction was further validated by blocking the activity of eprin/eprin receptor signaling during the differentiation of human iPSC towards cardiomyocytes. Such experimental validation further confirmed the predictability of novel regulatory genes/pathways in early human cardiovascular development using our multi-cell-fate comparison system. In addition to lineage-enriched protein-coding genes, we also applied the RNA-seq plus GEPA workflow to detect differentiation-associated RNA splicing isoforms and lineage-enriched lncRNAs during human cardiovascular differentiation (Fig. 7). A number of genes showing splicing switch during human cardiovascular differentiation were observed. Specifically, the progressive decrease of FGFR1 isoform with exon 3 and the emergence of FGFR1 isoform without exon 3 during MCP specification to cardiovascular lineages were validated, suggesting the change of FGF signaling sensitivity or differential intracellular cascades during pluripotency to cardiovascular specification. Moreover, we identified a number of lncRNAs with lineage-enriched expression pattern during human cardiovascular differentiation, such as a highly CM–specific long non-coding RNA LINC00881. This is consistent with a previous finding that LINC00881has the highest expression level in heart compared to other tissues^36^, implying that LINC0081 might have some unknown functions in human cardiomyocytes. Taken together, this study provided a new and comprehensive system to recapitulate and dissect early events of human heart formation, as well as to predict and validate novel regulatory genes, alternative RNA splicing, lncRNAs and mechanisms underling human cardiovascular development, which will benefit the basic research of mammalian heart development and the mechanistic study of human cardiovascular diseases.

## Methods

### Cell culture and cardiovascular differentiation

RUES2 human ES cells were obtained from Rockefeller University. Human Y1-iPS cells were reprogrammed from human dermal fibroblasts (HDF-α) (Cell Applications, USA) in our laboratory as previously described^14^. Human ES and iPS cells were maintained on MEFs and differentiated as described^14^. All growth factor were from R&D System. And chemicals were from Sigma Aldrich. Lithocholic acid was purchased from Sigma-Aldrich Co. LLC.

### Sample preparation and deep-transcriptome sequencing

Human RUES2, RUES2-derived-MCPs, cardiomyocytes, smooth muscle cells and endothelial cells were isolated and enriched as previously described^14^. Total RNAs were purified from the collected five cell samples using Qiagen RNeasy kit. Paired-end deep-transcriptome sequencing was performed using a service from LC Sciences (Houston, TX) with the Illumina platform.

### Sequencing data processing

Reads are mapped to human reference genome UCSC hg18 (ftp://igenome:G3nom3s4u @ftp.illumina.com/Homo_sapiens/UCSC/hg18) using Bowtie version 0.12.7 and Tophat version 1.3.2^37^. Expression intensity was estimated using Cufflinks version 1.2.1 as Fragment Per Kilobase of exon per Million reads (FPKM)^37^. Sum of all the isoforms with usable FPKM (quality status is “OK”) was used as the FPKM of each gene. Genes were removed if the FPKMs in the samples are all 0. After that, sum of the gene FPKM was calculated for each cell type. The value was then normalized to FPKM in 1 million of sum. Finally, we added 1 to all the normalized FPKM. Principle component analysis (PCA) and hierarchical clustering was performed using R (http://www.r-project.org/).

### Identification of the lineage-specific genes

The Gene Expression Pattern Analyzer (GEPA) algorithm was designed to recognize genes that were expressed at higher level in some lineages than in other lineages above a threshold of fold change. For each single gene, the lineage-specific expression pattern was recognized through the following steps. 1. Get the expression intensity values of each single gene in the samples (five samples in our study). 2. Sort the of the samples based on the gene expression values in descending order. 3. Compare the gene expression values in two samples as one cycle, starting the comparison of the highest value vs. the next highest. If the fold change is greater or equivalent to the threshold, stop and classify the gene with higher expression value as a specific-gene of that (those) sample(s) before the stopping point (not including the sample of lower value in current comparison). If the fold change is lower than the threshold, continue to compare the next two samples with the highest gene expression values. 4. If all the fold changes are less than the threshold, the expression pattern will be designated as “even” or “gradient” in all samples. If the fold change between the highest value and the lowest value is smaller than the threshold, the gene expression pattern will be designated as “even”. Otherwise the gene expression pattern will be referred to as “gradient”. In both “even” and “gradient” groups, fold change of gene expression level between any pair of samples that have closest FPKM values was below the threshold. The difference is that if the fold change between the maximal and minimal FPKM values is below the threshold the expression pattern would be recognized as “even”, while above the threshold would be a “gradient” pattern. For example, consider one gene that shows the highest FPKM in CM, second in MCP, third in SM, fourth in EC and lowest in ES and the threshold is set to 2.5. If the FPKM in CM is no less than 2.5 fold higher than MCP, the pattern will be recognized as CM specific. Otherwise, the algorithm will continue to compare MCP and SM. If MCP is no less than 2.5 fold higher than SM, the pattern will be MCP&CM specific. Otherwise, continue to compare next pairs of samples with closest FPKMs, such as SM and EC, until a pattern is designated. If the pattern cannot be designated in this way (that means all fold changes between two closest FPKMs are below 2.5), the FPKM in CM (highest) and ES (lowest) will next be compared. If the fold change is greater than 2.5, the pattern will be “gradient”, while lower than 2.5 would be “even”. We tested GEPA with thresholds of 1.5, 2.5 and 3.0 and set the threshold to 2.5 in all our analysis in this study because it has the lowest false positive plus false negative rates. List of housekeeping gene were downloaded from http://www.tau.ac.il/~elieis/HKG/. List of positive control gene sets were listed in Supplementary Table. 2. Lists of predicted cardiac development regulators based on histone modification markers and expression levels were from Paige *et al*.^13^. A Perl module implementing the GEPA algorithm can be accessed through http://sourceforge.net/projects/gepa/files/. Expression profiles of the genes in this study are provided in the Supplementary data sets.

### Function and canonical pathway enrichment analysis

Function and canonical pathway analyses were performed using Ingenuity IPA (http://www.ingenuity.com/products/pathways_analysis.html).

### Quantitative reverse transcription PCR (qRT-PCR) analysis

qRT-PCR was performed on a 7900HT Fast Real-Time PCR System (Applied Biosystems) with Fast SYBR Green Master Mix (Applied Biosystems). The results were analyzed using Excel, normalized to Cyclophinin gene expression, and compared to the undifferentiated ES cells. Primer sequences are described in Supplementary Table. 3. qRT-PCR results were presented as mean ± S.D. from three independent experiments in the Figures. Primers are listed in Supplementary Table. 3.

### Immunofluorescence Microscopy

The following antibodies were used: anti-human PECAM1 (CD31) (R&D System); anti-Cardiac Troponin T (CTNT) and anti-Smooth Muscle Actin (SMA) (Lab Vision); All secondary antibodies were from Life Technologies.

## Author Contributions

L.Y. and Y.L. designed the experiments and analysis; Y.L. and B.L. did the experiments; Y.L. did algorithm design and bioinformatical analysis; L.Y. and Y.L. wrote the manuscript. L.Y. oversaw the whole project.

## Additional Information

**How to cite this article**: Li, Y. *et al*. Comparative Transcriptomic Analysis of Multiple Cardiovascular Fates from Embryonic Stem Cells Predicts Novel Regulators in Human Cardiogenesis. *Sci. Rep.* **5**, 9758; doi: 10.1038/srep09758 (2015).

## Supplementary Material

Supplementary Information

Supplementary Video 1

Supplementary Video 2

Supplementary Dataset 1
