# Supplementary material for: Comparative Transcriptomic Analysis of Multiple Cardiovascular Fates from Embryonic Stem Cells Predicts Novel Regulators in Human Cardiogenesis
Source: Sci Rep. 2015 May 21;5:9758. doi: 10.1038/srep09758 (PMC4440522; doi:10.1038/srep09758)
Supplement: Supplementary Information [file srep09758-s1.doc]

**Tittle: Comparative Transcriptomic Analysis of Multiple Cardiovascular Fates from Embryonic Stem Cells Predicts Novel Regulators in Human Cardiogenesis**

**Authors and Affiliations:** Yang Li ^1^, Bo Lin ^1^, Lei Yang ^1, *^

^1^ Department of Developmental Biology, University of Pittsburgh School of Medicine, 530 45th Street, Rangos Research Center, Pittsburgh, PA 15201

***Correspondence information:**

Lei Yang, PhD

Department of Developmental Biology

University of Pittsburgh School of Medicine

530 45th Street, 8117 Rangos Research Center

Pittsburgh, PA 15201

Phone: 01-412-692-9842

Fax: 01-412-692-6184

Email: lyang@pitt.edu

**Supplementary Information**

**
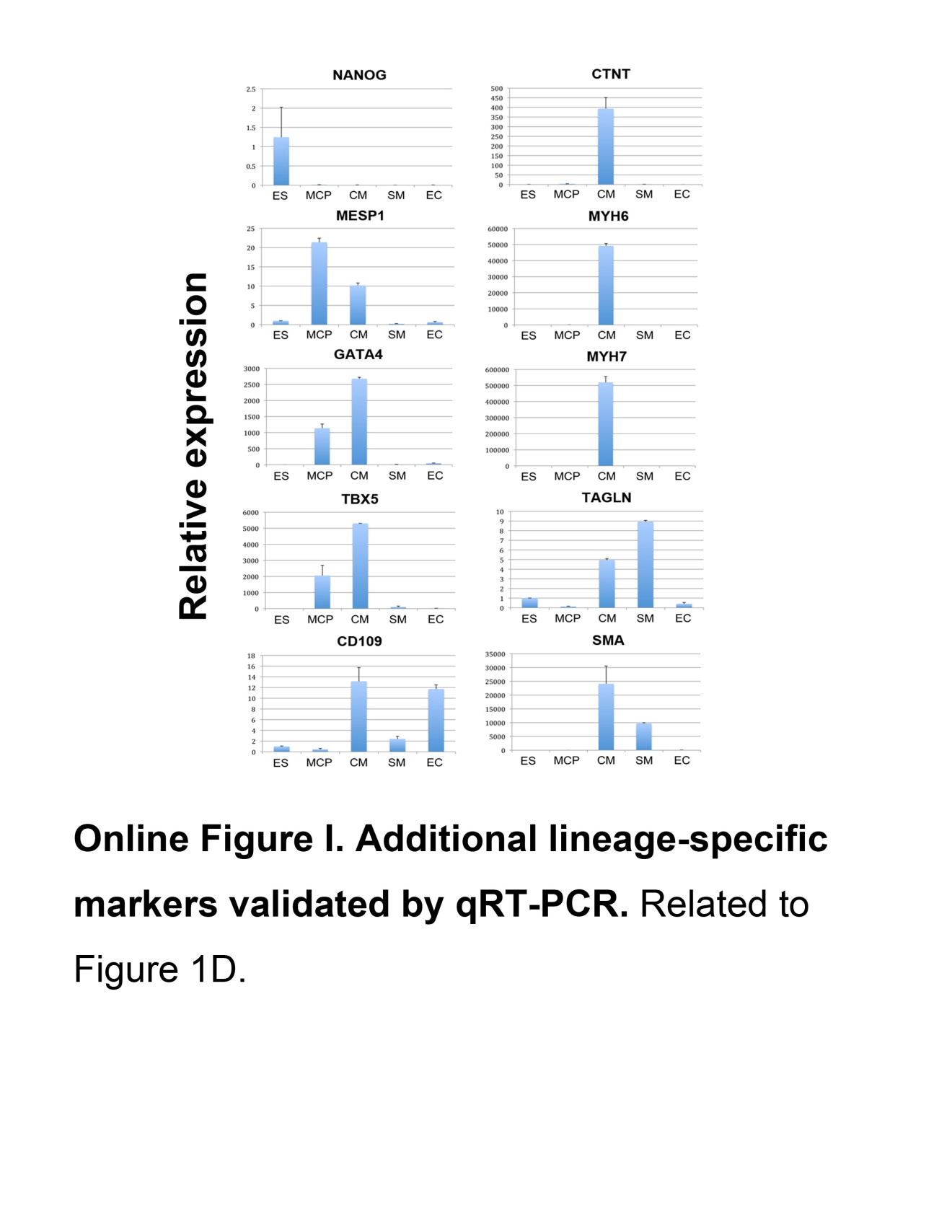
**

**Supplementary Figure. 1:** Additional lineage-specific markers validated by qRT-PCR**.** Related to Fig. 1d.

**
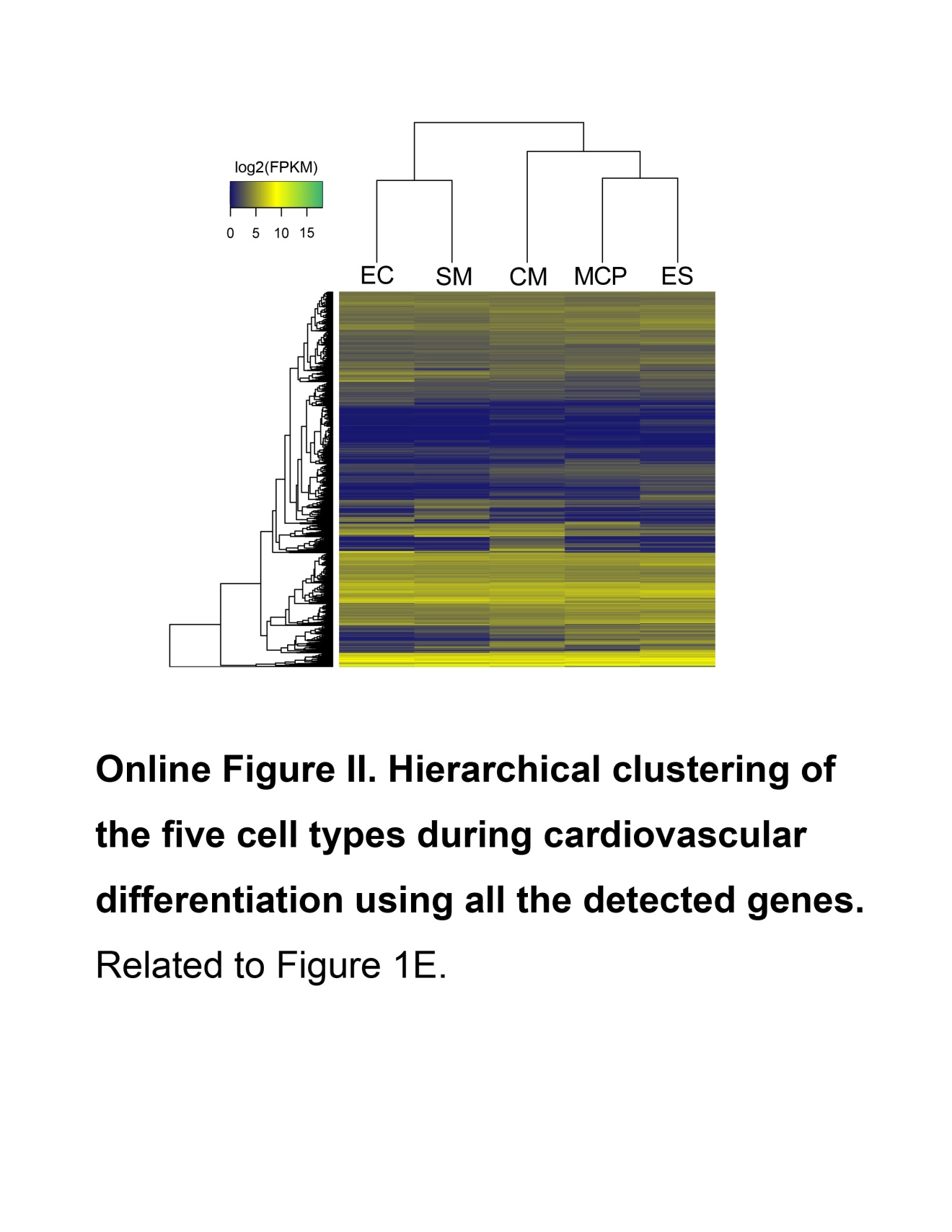
**

**Supplementary Figure. 2:** Hierarchical clustering of the five cell types during cardiovascular differentiation using all the detected genes. Related to Figure 1e.


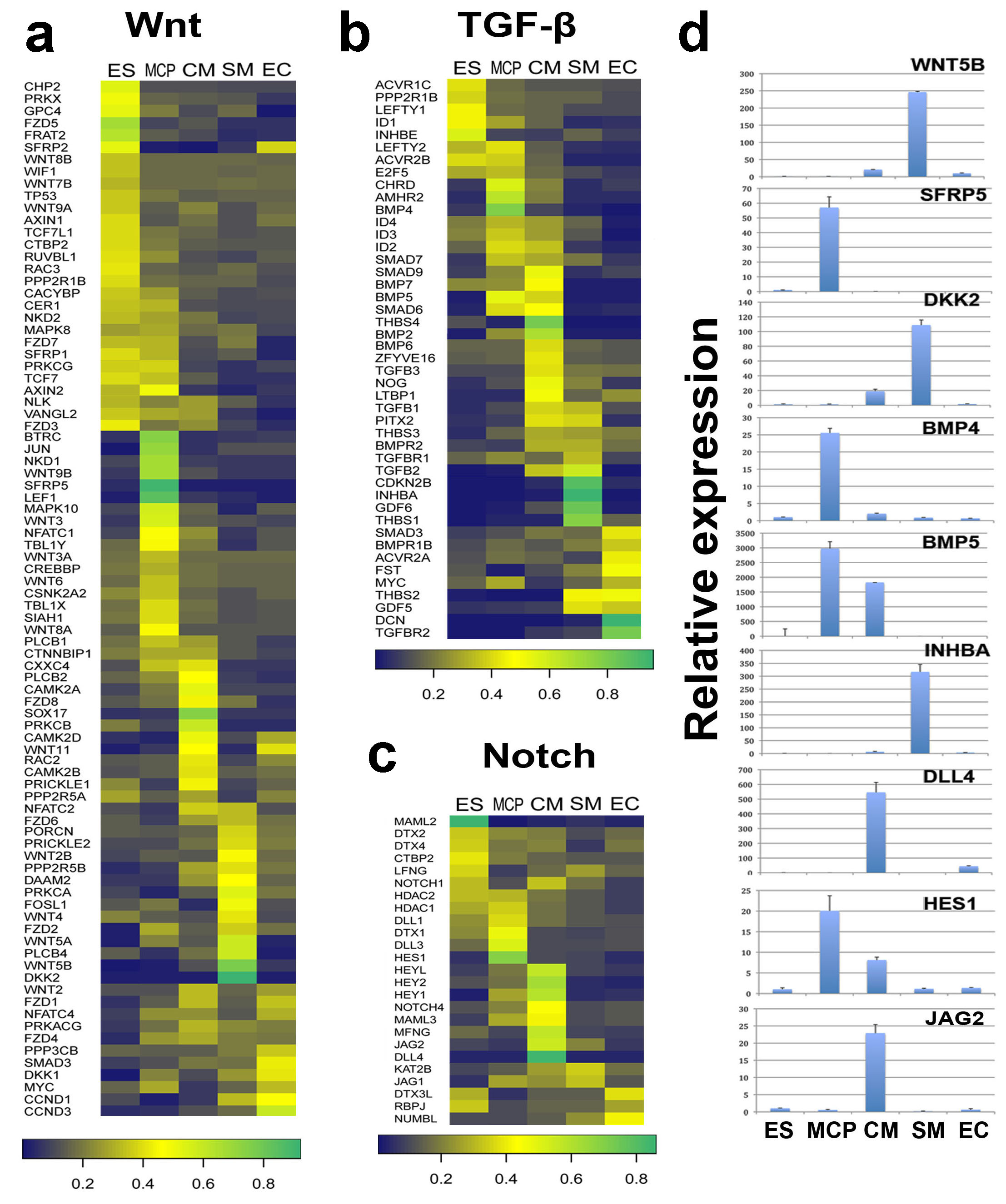


**Supplementary Figure. 3**: Dynamic expressions of pathway components during the cardiovascular differentiation. To indicate the lineage-specificity, the relative gene expression percent in the sum of all the cell types was shown in the heatmap. (a) Genes in Wnt signaling pathway. (b) Genes in TGF-β signaling pathway. (c) Genes in Notch signaling pathway. (d) qRT-PCR validation of the expression pattern of above signaling pathway component genes in the five cell types.

**Supplementary Video 1.** Day 20 embryoid bodies differentiated from hiPSC with DMSO in medium from day 4 to day 20.

**Supplementary Video 2.** Day 20 embryoid bodies differentiated from hiPSC with 200 μM lithocholic acid in medium from day 4 to day 20.

| **Supplementary Table 1**. Positive control sets used to estimate false negative rates of GEPA predictions | |
| --- | --- |
| Gene | Cell type |
| ACTN2 | cardiomyocytes |
| NPPA | cardiomyocytes |
| NPPB | cardiomyocytes |
| SGCA | cardiomyocytes |
| GJA4 | cardiomyocytes |
| GJA5 | cardiomyocytes |
| FABP3 | cardiomyocytes |
| GATA4 | cardiomyocytes |
| GATA6 | cardiomyocytes |
| HCN4 | cardiomyocytes |
| MEF2C | cardiomyocytes |
| MYH7 | cardiomyocytes |
| MYH6 | cardiomyocytes |
| MYH11 | cardiomyocytes |
| TNNT2 | cardiomyocytes |
| TNNI3 | cardiomyocytes |
| TNNI1 | cardiomyocytes |
| TBX2 | cardiomyocytes |
| TBX3 | cardiomyocytes |
| TBX5 | cardiomyocytes |
| PLN | cardiomyocytes |
| MYL7 | cardiomyocytes |
| MYL2 | cardiomyocytes |
| HAND2 | cardiomyocytes |
| NKX2-5 | cardiomyocytes |
| ISL1 | cardiomyocytes |
| MYOCD | cardiomyocytes |
| TBX20 | cardiomyocytes |
| MYBPC3 | cardiomyocytes |
| ATP2A2 | cardiomyocytes |
| NANOG | embryonic stem cell |
| SOX2 | embryonic stem cell |
| POU5F1 | embryonic stem cell |
| GDF3 | embryonic stem cell |
| ZFP42 | embryonic stem cell |
| DNMT3B | embryonic stem cell |
| KLF4 | embryonic stem cell |
| TERT | embryonic stem cell |
| FGF4 | embryonic stem cell |
| DPPA5 | embryonic stem cell |
| DPPA2 | embryonic stem cell |
| DPPA4 | embryonic stem cell |
| TDGF1 | embryonic stem cell |
| LEFTY1 | embryonic stem cell |
| UTF1 | embryonic stem cell |
| LEFTY2 | embryonic stem cell |
| GRB7 | embryonic stem cell |
| PODXL | embryonic stem cell |
| CD9 | embryonic stem cell |
| GAL | embryonic stem cell |

| Supplementary Table 2. Distribution of genes in lineage-specific categories classified by GEPA and comparison to random shuffle | | | | | | |
| --- | --- | --- | --- | --- | --- | --- |
|  | Original | | | Shuffle | | |
| Pattern | 1.5 | 2.5 | 3 | 2.5 | 2.5 | 2.5 |
| ES | 1683 | 472 | 346 | 1457 | 1437 | 1441 |
| MCP | 693 | 211 | 167 | 1280 | 1285 | 1296 |
| CM | 996 | 368 | 291 | 1396 | 1361 | 1382 |
| SM | 675 | 263 | 201 | 1270 | 1299 | 1241 |
| EC | 902 | 366 | 286 | 1373 | 1378 | 1352 |
| ES MCP | 769 | 228 | 118 | 288 | 278 | 303 |
| ES CM | 137 | 40 | 27 | 315 | 355 | 338 |
| ES SM | 71 | 21 | 11 | 240 | 253 | 279 |
| ES EC | 87 | 17 | 12 | 258 | 238 | 289 |
| MCP CM | 209 | 92 | 77 | 327 | 327 | 284 |
| MCP SM | 24 | 8 | 3 | 244 | 236 | 257 |
| MCP EC | 19 | 12 | 8 | 260 | 253 | 234 |
| CM SM | 143 | 61 | 48 | 287 | 269 | 301 |
| CM EC | 104 | 46 | 30 | 282 | 267 | 289 |
| SM EC | 375 | 136 | 108 | 223 | 221 | 224 |
| ES MCP CM | 508 | 242 | 176 | 284 | 276 | 266 |
| ES MCP SM | 17 | 3 | 3 | 175 | 201 | 171 |
| ES MCP EC | 131 | 9 | 4 | 172 | 181 | 161 |
| ES CM SM | 44 | 17 | 16 | 228 | 216 | 260 |
| ES CM EC | 27 | 2 | 1 | 219 | 218 | 226 |
| ES SM EC | 65 | 17 | 9 | 177 | 161 | 166 |
| MCP SM EC | 15 | 1 | 1 | 154 | 153 | 148 |
| MCP CM SM | 58 | 26 | 23 | 224 | 210 | 217 |
| MCP CM EC | 47 | 12 | 9 | 183 | 213 | 224 |
| CM SM EC | 358 | 139 | 100 | 182 | 171 | 185 |
| ES MCP CM SM | 199 | 125 | 102 | 412 | 438 | 453 |
| ES MCP CM EC | 173 | 50 | 35 | 411 | 404 | 407 |
| ES MCP SM EC | 25 | 4 | 2 | 273 | 274 | 238 |
| ES CM SM EC | 164 | 69 | 47 | 322 | 352 | 319 |
| MCP CM SM EC | 244 | 129 | 96 | 292 | 311 | 297 |
| Gradient | 4500 | 4683 | 3898 | 1795 | 1780 | 1751 |
| Even | 1670 | 7263 | 8877 | 129 | 116 | 133 |

| Supplementary Table 3. Primers for qRT-PCR | |
| --- | --- |
| gene | sequence |
| NANOG-F | CAGAAGGCCTCAGCACCTAC |
| NANOG-R | GTCACTGGCAGGAGAATTTGG |
| POU5F1-F | AACCTGGAGTTTGTGCCAGGGTTT |
| POU5F1-R | TGAACTTCACCTTCCCTCCAACCA |
| MESP1-F | GATGGAGCCAAGCCCAC |
| MESP1-R | TCAGTTGTCCCTTGTCACTTG |
| MSX1-F | TCCAGTTTCACCTCTTTGCTCCCT |
| MSX1-R | TTAACCTCTCTGCCCTCAGTTTCCC |
| ISL1-F | TTGTACGGGATCAAATGCGCCAAG |
| ISL1-R | AGGCCACACAGCGGAAACA |
| HAND2-F | CGCCGACACCAAACTCTCCAAA |
| HAND2-R | GCTCCTTCTTCCTCTTCTCCTCTTTCAC |
| GATA4-F | GGAAGCCCAAGAACCTGAAT |
| GATA4-R | GTTGCTGGAGTTGCTGGAA |
| TBX20-F | GTCTCTCAGCTCCTGGGTATCATCTT |
| TBX20-R | TGTTGCTATGGATGCTGTGCTGGT |
| TBX5-F | AAATGAAACCCAGCATAGGAGCTGGC |
| TBX5-R | ACACTCAGCCTCACATCTTACCCT |
| NKX2.5-F | ACCTCAACAGCTCCCTGACTCT |
| NKX2.5-F | ATAATCGCCGCCACAAACTCTCC |
| CTNT-F | AAAGCCCAGGTCGTTCATGCCC |
| CTNT-R | CATTCCGGATGCGCTGCTGC |
| MYH6-F | GGGATAACCAGGGGAAGCACCAAGA |
| MYH6-R | TGCCTCCCTCCCGGGACAAAT |
| MYH7-F | TTGAGTAGCCCAGGCACAGCCAT |
| MYH7-R | TGCCACCCTCTCGAGACACGAT |
| SMA-F | AATACTCTGTCTGGATCGGTGGCT |
| SMA-R | ACGAGTCAGAGCTTTGGCTAGGAA |
| CNN1-F | ACAACCACCACGCACACAACTAC |
| CNN1-R | GTCTGCTCTCTCCAAACTCTAACCCT |
| TAGLN-F | CTGACATGTTCCAGACTGTTGACCTCTT |
| TAGLN-R | CCTCTTATGCTCCTGCGCTTTCTT |
| CD109-F | TGCCCAGTGTATTGAATTGGTGGTAGA |
| CD109-R | ACCATACAGGCTTGAAGGACCAGAAC |
| LAMA4-F | CTCCTAGCCTGGGCAACATAATGAGA |
| LAMA4-R | AGTGCAGGTACAGTCAGCAGTCATAG |
| LIN28A-F | GTGAGGCAGTGGAGTTCACCTTT |
| LIN28A-R | TCTGCGCTTCTGCATGCTCTTT |
| FOXH1-F | ACTCCATTCGCCACAACCTTTCCT |
| FOXH1-R | CGCCCAGAAGTTGCCCTTG |
| DNAH2-F | TAGAGAAAGGCATCCAGGGTCTCATC |
| DNAH2-R | CCACAGCTCAAACTGCTCCACA |
| LIX1-F | CCCGACAGGAAGTCATCTCCTACTATTC |
| LIX1-R | TTCTTGTCCTGCTTTCCTGGCTTC |
| PDLIM3-F | AAGTGTTCTCCTCCCATAAGGTCCCA |
| PDLIM3-R | ACAGGTCAGGGTCAGAGTCAGTT |
| HDAC9-F | TGAACGGATCGTAATTCCACCCAGA |
| HDAC9-R | GCCTCATGCTAGTTCCTCCATCTTGAAC |
| GREM1-F | CCTCCTCCTCACAATCCATCTCTTCT |
| GREM1-R | ACCCTCCCTTTCTCACTCCACTATC |
| CDKN2A-F | CATGGAGCCTTCGGCTGACT |
| CDKN2A-R | CCGTAACTATTCGGTGCGTTGGG |
| RUNX1-F | GAGAAATGCTACCGCAGCCATGAA |
| RUNX1-R | ACTTGCGGTGGGTTTGTGAAGA |
| SIX2-F | GCCAAGGAAAGGGAGAACAACGAGAA |
| SIX2-R | TCATCCTCCGAGCTGCCTAACA |
| BICC1-F | AGAACGCCCACAAATACCTGGAGTG |
| BICC1-R | TTGGACCGTGAAAGGGACATGGA |
| HHIP-F | TGTCGTCATGGAGGTGTCTGTGTTAG |
| HHIP-R | TGGTCACTCTGCGGATGTTTCTGT |
| MEIS2-F | GCCACCGATACATTAGCTGTTTGAAGGG |
| MEIS2-R | GGTTATGGTCAGCGAGATTTGTGGAG |
| SNAI2-F | CAACAGAGCATTTGCAGACAGGTCAA |
| SNAI2-R | GCTACACAGCAGCCAGATTCCTC |
| FZD5-F | ATCATCTTCCTGTCAGCCTGCTACCT |
| FZD5-R | TGGTCTCGTAGTGGATGTGGTTGTG |
| SFRP5-F | ACCGCTGGGACAAGAAGAATAAGGAG |
| SFRP5-R | CGTAGAAGAAAGGGTAGTAGAGGGAGCA |
| WNT5B-F | TACCGCTTCGCCAAGGAGTTTGT |
| WNT5B-R | TTTGCAGGCTACGTCTGCCATCTT |
| DKK2-F | GCCAAACTCAACTCCATCAAGTCCTCTC |
| DKK2-R | TGTGGCAATACCTCCCAACTTCAC |
| WNT11-F | CTGTGAAGGACTCGGAACTCGTCTATCT |
| WNT11-R | TGTCTTGTTGCACTGCCTGTCTTG |
| BMP4-F | ATCAGGAGATGGTAGTAGAGGGATGTGG |
| BMP4-R | GTGGTGTATGTGGTGTGTGTGTGT |
| BMP7-F | TGTGCCTTCCCTCTGAACTCCTAC |
| BMP7-R | CCCGGACCACCATGTTTCTGTATTTCT |
| BMP5-F | CGTGAGAGCAGCCAACAAACGAAA |
| BMP5-R | TGGTGCTATAATCCAGTCCTGCCATC |
| TGFB2-F | GCTTTGGATGCGGCCTATTGCTTT |
| TGFB2-R | GGCATTGTACCCTTTGGGTTCGTGTATC |
| TGFBR2-F | CCTGTGTCGAAAGCATGAAGGACAAC |
| TGFBR2-R | CTCAGTCAACGTCTCACACACCATCT |
| INHBA-F | CAGTTTCAAGGACATCGGCTGGAATG |
| INHBA-R | TGTTGAGTGGAAGGACAGTGAGGA |
| HES1-F | GCCAGTTTGCTTTCCTCATTCCCA |
| HES1-R | TCCACATGGAGTCCGCCGTAA |
| JAG2-F | GGAGGATGAGGAGGACGAGGA |
| JAG2-R | CGGCCAGGATCTTTGGTGAATTTGTG |
| DLL4-F | CAGCAGGGAAGCCATGAACAACT |
| DLL4-R | AGTCCACTTCCAGCTCCTTCTTCT |
| MFNG-F | GGGAAACTCAACGTCATTAAGCTACAGGG |
| MFNG-R | TGGATAGAGCAGACAATGGAGGGAG |
| CYCLOPHININ-F | GAAGAGTGCGATCAAGAACCCATGAC |
| CYCLOPHININ-R | GTCTCTCCTCCTTCTCCTCCTATCTTTACTT |
| FGFR1-primer1-F | CCTTGTCACCAACCTCTAAC |
| FGFR1-primer1-R | GATGTCCAATATGGAGCTACG |
| FGFR1-primer2-F | GATGTGCAGAGCATCAACTG |
| FGFR1-primer2-R | CTGGTTACGCAAGCATAGAG |
| NR_034008-F | CAGGGCCTTTGAAGAAGTAA |
| NR_034008-R | TGTGCATCTGTGTCCTAATC |
| NR_028406-F | GAAGAGGCCTCCTATCTACTC |
| NR_028406-R | CACTCCTGAAGCAGAGAAAC |
| NR_021490-F | CGAGGATCTTGGGATCATTTC |
| NR_021490-R | CTGGCCCTCATTAGGATTTAC |
